# Call to Action on World Pneumonia Day

**DOI:** 10.3201/eid1811.121217

**Published:** 2012-11

**Authors:** Rana Hajjeh, Cynthia G. Whitney

**Affiliations:** Author affiliation: Centers for Disease Control and Prevention, Atlanta, Georgia, USA

**Keywords:** pneumonia, health care, World Health Organization, WHO, World Pneumonia Day, bacteria, Global Action Plan for Prevention and Control of Pneumonia, Global Alliance for Vaccines and Immunizations

This month, on November 12, the world will recognize the fourth annual World Pneumonia Day. First launched in 2009 by a coalition of global health leaders (*1*), World Pneumonia Day aims to raise awareness about pneumonia’s toll on the world’s children and to promote interventions to protect against, treat, and prevent the disease. Pneumonia continues to be the leading killer of young children around the world, causing ≈14% of all deaths in children 1 month to 5 years of age (*2*). It is a critical disease for countries to conquer in order to reach Millennium Development Goal 4: reducing the child mortality rate by two thirds from 1990 to 2015 (*3*). Most children who die from pneumonia live in developing countries, where such factors as malnutrition, crowding, and lack of access to quality health care increase the risk for death. Pneumonia kills few children in industrialized countries, although it remains among the top 10 causes of deaths in the United States, for example, because of deaths in older adults (*4*).

Fortunately, many interventions are now available to reduce deaths due to pneumonia among children throughout the world. On the first World Pneumonia Day in 2009, the World Health Organization and the United Nations Children’s Fund, together with many global experts and partners, launched the Global Action Plan for Prevention and Control of Pneumonia (GAPP) (*5*). GAPP recommends a strategy of prevention, protection, and treatment that is designed to implement readily available interventions that can reduce pneumonia deaths in children. GAPP focuses on improving nutrition (through measures such as exclusive breastfeeding), increasing access to vaccines that protect from agents that cause pneumonia (such as *Haemphilus influenzae* type b and pneumococcal vaccines), reducing exposure to indoor air pollution, and increasing access to antimicrobial drugs that can treat pneumonia.

In 2010, the World Health Assembly passed a resolution recognizing the role of pneumonia as the leading cause of deaths in children, setting out the goal of reducing pneumonia deaths as a global health priority (*6*), and the World Health Organization began tracking implementation of GAPP. One notable area of success has been the introduction of new vaccines to prevent pneumonia. During the last few years, because of funding and technical support from the Global Alliance for Vaccines and Immunizations and various partners, the introduction of new vaccines in developing countries has had unprecedented momentum. *Haemophilus influenzae* type b vaccines have been introduced or are ready to be introduced in all countries eligible for Global Alliance for Vaccines and Immunizations funding by 2013, and pneumococcal conjugate vaccines are expected to be introduced in 54 countries by 2015 (*7*).

Despite recent progress in the effort to decrease the number of pneumonia cases, pneumonia is still an urgent problem. This month’s issue of Emerging Infectious Diseases presents results of recent research on pneumonia conducted around the world. The work, mostly from high-income settings, highlights some of the remaining difficulties involving pneumonia prevention, treatment, and control. For example, although van Deursen et al. show the remarkable benefits of the pneumococcal conjugate vaccination program in the Netherlands (*8*), Fleming-Dutra et al. report how pneumococcal pneumonia outbreaks can occur even in a highly vaccinated population if crowding and poor health are common, because currently available vaccines do not cover all pneumococcal serotypes (*9*). By describing cases of pneumonia that occurred after the megaquake in Japan in 2011, Takahashi et al. show how natural disasters might lead to increases in pneumonia risk or create large shifts in needed health care (*10*).

Many challenging research questions remain. A recent priority-setting exercise outlined the most urgent studies needed to reduce pneumonia deaths in low-income countries (*11*). Priority items ranged from assessments of vaccine effects on disease in low-income settings to evaluation of measures to improve community management of pneumonia. In addition, a large, multicenter study to identify the etiologic agents of pneumonia in developing countries, supported by the Bill and Melinda Gates Foundation, was launched in 2011 (*12*). This study is expected to generate data that will better guide prevention and treatment strategies, particularly in countries that are already using new vaccines.

This November 12, World Pneumonia Day, we urge the global community to consider the massive problems of pneumonia. Better yet, take a moment to consider what you can do to solve this problem. Health care providers, researchers, policy makers, and the greater public health community all need to contribute if we are to make rapid, substantial progress toward reducing disease and deaths due to pneumonia. Progress is being made, but much more can be done.
